# Low Oxygen Tension Enhances Expression of Myogenic Genes When Human Myoblasts Are Activated from G_0_ Arrest

**DOI:** 10.1371/journal.pone.0158860

**Published:** 2016-07-21

**Authors:** Jeeva Sellathurai, Joachim Nielsen, Eva Kildall Hejbøl, Louise Helskov Jørgensen, Jyotsna Dhawan, Michael Friberg Bruun Nielsen, Henrik Daa Schrøder

**Affiliations:** 1 Institute of Clinical Research, SDU Muscle Research Cluster (SMRC), University of Southern Denmark, Odense, Denmark; 2 Department of Sports Science and Clinical Biomechanics, SDU Muscle Research Cluster (SMRC), University of Southern Denmark, Odense, Denmark; 3 Institute for Stem Cell Biology and Regenerative Medicine (inStem), National Center for Biological Sciences, Bangalore, India; 4 Department of Clinical Pathology, Odense University Hospital, Odense, Denmark; University of Minnesota Medical School, UNITED STATES

## Abstract

**Objectives:**

Most cell culture studies have been performed at atmospheric oxygen tension of 21%, however the physiological oxygen tension is much lower and is a factor that may affect skeletal muscle myoblasts. In this study we have compared activation of G_0_ arrested myoblasts in 21% O_2_ and in 1% O_2_ in order to see how oxygen tension affects activation and proliferation of human myoblasts.

**Materials and Methods:**

Human myoblasts were isolated from skeletal muscle tissue and G_0_ arrested *in vitro* followed by reactivation at 21% O_2_ and 1% O_2_. The effect was assesses by Real-time RT-PCR, immunocytochemistry and western blot.

**Results and Conclusions:**

We found an increase in proliferation rate of myoblasts when activated at a low oxygen tension (1% O_2_) compared to 21% O_2_. In addition, the gene expression studies showed up regulation of the myogenesis related genes *PAX3*, *PAX7*, *MYOD*, *MYOG* (myogenin), *MET*, *NCAM*, *DES* (desmin), *MEF2A*, *MEF2C* and *CDH15* (M-cadherin), however, the fraction of DES and MYOD positive cells was not increased by low oxygen tension, indicating that 1% O_2_ may not have a functional effect on the myogenic response. Furthermore, the expression of genes involved in the TGFβ, Notch and Wnt signaling pathways were also up regulated in low oxygen tension. The differences in gene expression were most pronounced at day one after activation from G_0_-arrest, thus the initial activation of myoblasts seemed most sensitive to changes in oxygen tension. Protein expression of HES1 and β-catenin indicated that notch signaling may be induced in 21% O_2_, while the canonical Wnt signaling may be induced in 1% O_2_ during activation and proliferation of myoblasts.

## Introduction

Normal metabolism and function of cells are dependent on a continuous and regulated supply of oxygen, and if the oxygen levels are reduced due to pathophysiological conditions, the metabolic and cellular functions are altered [1]. Physiological oxygen tension in skeletal muscle tissue has been reported to range between 1–10% [1–4], while oxygen tensions less than 1% has been suggested to reflect conditions of physiological hypoxia [4], however there are some discrepancies on exactly at what oxygen tension physiological hypoxia occurs.

A vast majority of *in vitro* cell culture studies have been conducted using atmospheric conditions (i.e. 21% O_2_). However, the hypothesis that lower oxygen levels in cell cultures would reflect the in *vivo* conditions better has sparked a number of myoblast culture studies using low oxygen tension (Table 1) ranging from 0.5–6% and employing cells from different species, which has led to different results on cell proliferation and differentiation.

**Table 1:** Overview of myogenic cell culture studies conducted with different oxygen tensions.

**Study**	**Species**	**Oxygen tension**	**Effects of different oxygen tension on myoblasts (compared to 21%)**	**Publication year**
Kook S.H. et al [5]	Bovine myoblasts	1%	Proliferation ↑ Differentiation ↑	2008
Launay T. et al [3]	Rat L6 myoblast cell line	1%	Proliferation ↓ Differentiation↓	2010
Hidalgo M. et al [6]	Rat L6 myoblast cell line	1. 1%	1. Proliferation ↓ Differentiation ↓	2014
		2. 5%	2. No difference in proliferation compared to 21% O_2_. Differentiation ↑	
Lees S.J. et al [7]	Rat primary myoblasts	5%	Proliferation ↑	2008
Chakravarthy MV et. al [2]	Aged rat myoblast	3%	Proliferation ↑ Myotube size ↑	2001
Urbani L. et al [8]	Mouse primary isolated myoblast	2%	Proliferation ↑ Differentiation ↑	2012
Konigsberg M. et al [9]	Mouse primary isolated myoblasts	3%	Proliferation after 5 days in culture ↑ Differentiation ↑	2013
Csete M. et al [10]	Mouse Single fiber isolation	6%	Proliferation ↑ Survival of mature fibers↑	2001
Di C.A. et al [11]	C2C12	1%	Proliferation ↓ Differentiation ↓	2004
Kook S.H. et al [5]	C2C12 cell line	1%	Differentiation ↓	2008
Yun Z. et al [4]	C2C12	0,5%	Transient reduction in differentiation. Cells adapt to low oxygen level and differentiate after 6–12 days.	2005
Launay T. et al[3]	Human myoblasts	1%	1–3 days after culture: no change in proliferation. 4 days after culture: proliferation ↓ Differentiation ↓	2010
Koning M. et al [12]	Human myoblasts	2%	Proliferation for the first five passages ↑	2011
Martin S.D. et al [13]	Human myoblasts	5%	3 days after culture: Proliferation↑ No difference in differentiation	2009

Employing a reduced oxygen tension of 5% resulted in an increase in proliferation of primary isolated rat myoblasts [7], however the same oxygen tension did not increase proliferation in rat L6 myoblasts [6]. An oxygen tension of 3% resulted in a significant increase in proliferation and differentiation of primary isolated mouse myoblasts [9] and in myoblast cultures from aged rats [2]. An oxygen tension of 2% led to increased mouse myoblast proliferation and differentiation, too [8]. Furthermore, cultures of single myofibers isolated from mice showed that both satellite cell proliferation and survival of mature fibers increased when reducing oxygen tension from 21% to 6% O_2_ [10]. Recently, exposing bovine satellite cell cultures to 1% oxygen tension was shown to stimulate proliferation and differentiation, and the low oxygen tension appeared to up regulate the Myogenic Regulatory Factors (MRFs) [5]. In contrast, other studies have reported impaired proliferation and inhibited myogenic differentiation of C2C12 myoblasts when cultured at 1% O_2_ levels [5,11]. However, in a study by Yun et al. the reduction in differentiation of C2C12 myoblasts in 0.5% O_2_ was transient, as cells cultured for 6–12 days were able to adapt to chronic low oxygen tension and still form myotubes [4].

The effect of low oxygen tension (1% O_2_) has recently been studied in primary isolated human myoblasts, and compared to normoxia (21% O_2_), no change in proliferation rate was found initially, but after 4 days in culture the proliferation rate was reduced when cultured in low oxygen tension [3]. Moreover, the differentiation was significantly impaired [3]. In contrast, two studies reported an increase in proliferation rate of human myoblasts cultured in 2% and 5% O_2_, respectively [12,13].

Thus, in most studies, oxygen tension levels between 2–6% have led to significant increases in proliferation of myoblasts in all species examined (Table 1). In contrast, 1% oxygen tension seem to represent a borderline between normal physiologic and hypoxic conditions, since some studies have reported increased proliferation of myoblasts while others observed decreased proliferation (Table 1). Satellite cells are normally activated after muscle damage, and in that context the cells may experience lower than physiologic oxygen tension because of the decreased blood circulation.

Studies on the effects of O_2_ are important given that lower *in vitro* O_2_ conditions could provide more comparable results to *in vivo* conditions, and optimized *in vitro* O_2_ conditions could be of value for isolation and propagation of myoblasts for clinical use. In that context it is important to study the behavior of particularly human myoblasts in oxygen tension closer to the *in vivo* state.

In the present study we investigated the effect of low oxygen tension (1% O_2_) on primary isolated human myoblasts. While other cell culture studies performed at low oxygen tension have mainly focused on myoblast proliferation and differentiation, we here focus on activation and proliferation of G_0_ arrested myoblasts in 1% O_2_ using our recently published model for inducing *in vitro* G_0_-arrest [14]. This model allowed us to study a synchronized activation of myoblast cultures, corresponding to conditions mimicking activation *in vivo*.

## Results

### Isolation of myoblasts and purity of the cultures

**Fig 1:**
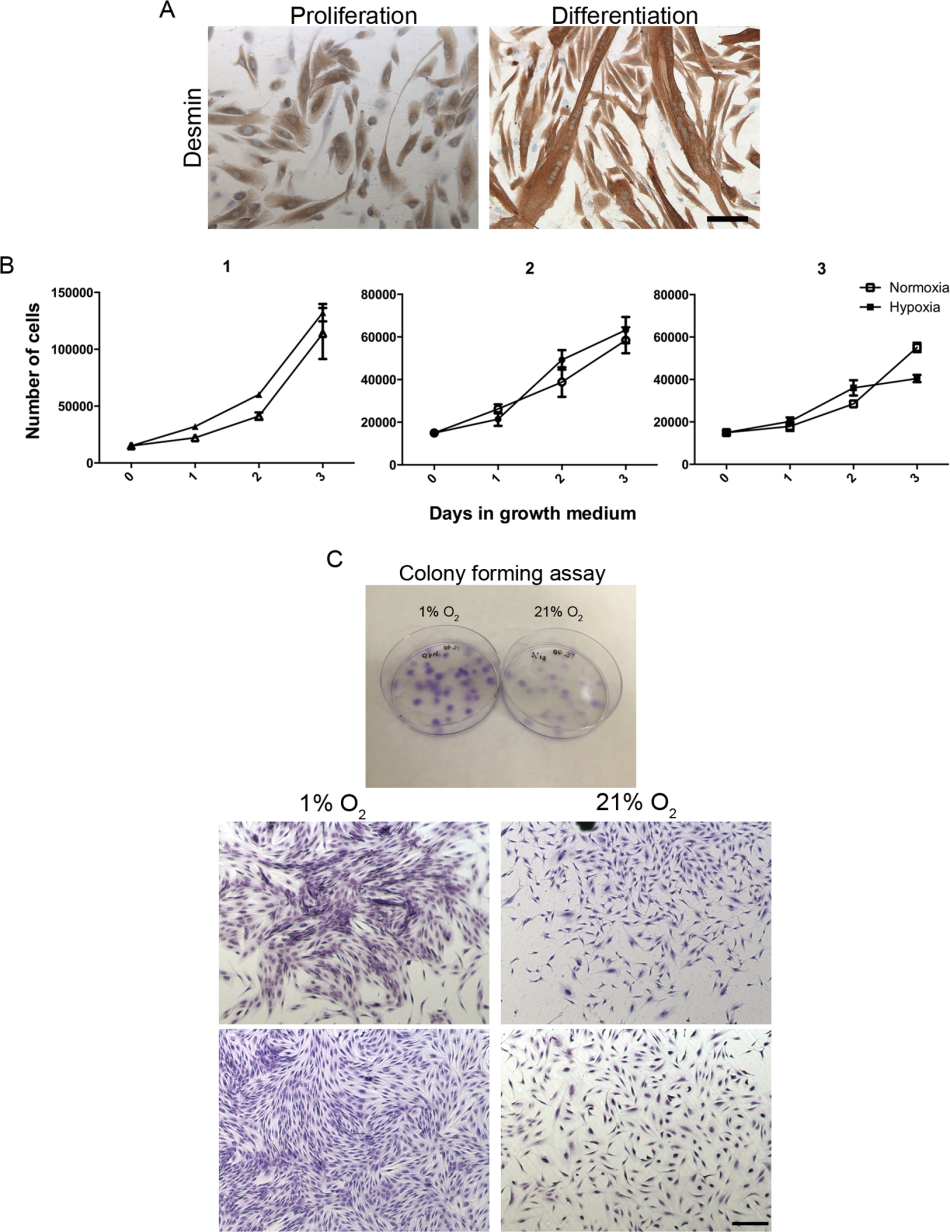
Myoblast purity and proliferation rate. The purity of the isolated myoblasts was tested by desmin stainings and differentiation assays (A). Almost all of the isolated cells were desmin positive confirming a high level of myoblast purity and the myoblasts were able to form large multinucleated myofiber when cultured in differentiation medium. Proliferation rate of myoblasts in 1% O_2_ and 21% O_2_, respective, was measured by proliferation assays (three days) and colony forming assays (14 days). The short-term proliferation rate demonstrated no difference in myoblast proliferation (B). The colony forming assays (crystal violet staining) (C) demonstrated no difference in the number of colonies formed by myoblasts in 1% and 21% O_2_, however, the colonies formed in 1% O_2_ were bigger and had a higher cell density, thus demonstrating an induced proliferation. Scale bar: 400 μm.

Human myoblasts were isolated from muscle biopsies, cultured *in vitro* and immunostained for the expression of desmin (DES) (Fig 1A). Almost all of the isolated cells were desmin positive and the cells were able to differentiate and form large myofibers when cultured in differentiation medium, confirming that the used isolation method resulted in a highly purified satellite cell population. The myoblasts were used within 5–6 passages to ensure a sufficient purity level.

### Proliferation of human myoblasts was induced by low oxygen tension

The effect of low oxygen tension (1% O_2_) on primary isolated human myoblasts was evaluated with cells from skeletal muscle biopsies obtained in 3 young subjects. The cells were upscaled in 21% O_2_ and divided into two fractions, of which one was cultured in 21% O_2_ and the other in low oxygen tension (1% O_2_). Proliferation rate of the myoblasts was measured by proliferation assays lasting three days (Fig 1B) and colony forming assays lasting 14 days (Fig 1C). The short-term proliferation rates displayed some individual variation in growth between the tree cultures, however, no significant difference in growth rate was observed between the two culture conditions (Fig 1B). In the colony forming assays visualized by crystal violet staining we found no difference in the number of colonies formed by myoblasts in 1% and 21% O_2_ (data not shown), however, the colonies formed in 1% O_2_ were more densely populated (Fig 1C). Thus long-term myoblast proliferation was induced at 1% O_2_.

### Low oxygen tension enhances expression of several genes in human myoblasts during activation from G_0_ arrest

We have previously described a model for inducing G_0_-arrest in human myoblasts by keeping the cells in a viscous gel thus depriving cell attachment [14]. A similar model has been used to verify cell cycle arrest in mouse myoblasts and fibroblasts [15–20]. Using this protocol we induced G_0_-arrest in myoblast cultures from the three subjects. The induction of G_0_ was made at 21% O_2_ to secure sufficient O_2_ diffusion in the high viscosity gel. The G_0_ arrested cells were afterwards reactivated in growth medium, immediately divided into two fractions, of which one was cultured at 21% O_2_, whereas the other fraction was cultured at 1% O_2_ for three days. Samples were collected each day for immunocytochemistry, realtime RT-PCR analysis and western blots.

### The cell cycle regulatory genes

**Fig 2:**
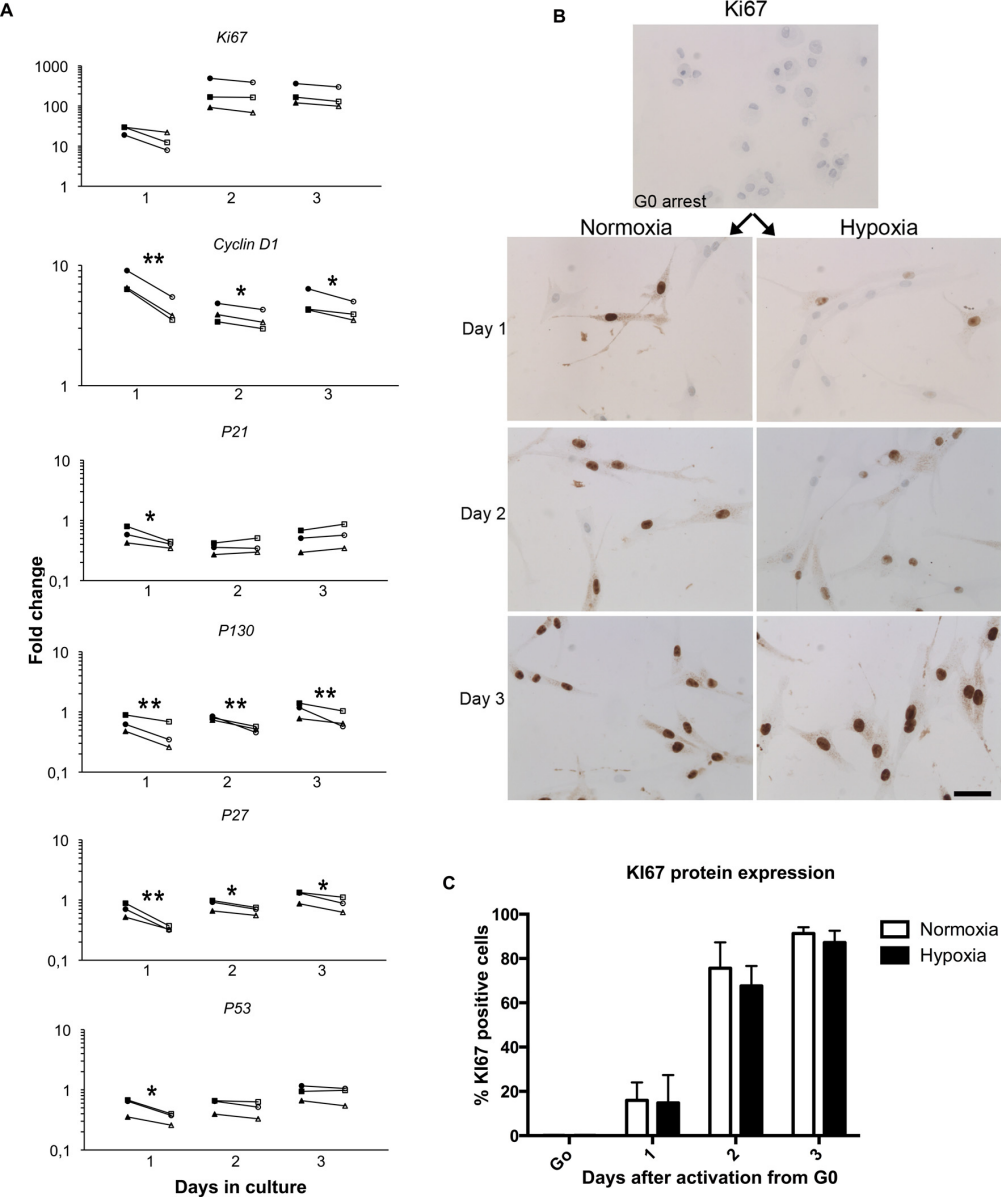
Expression of cell cycle related genes and KI67 protein expression in human myoblast cultures were studied at 1% O_2_ and 21% O_2_. The expressions of the cell cycle related genes *P27*, *CCND1*, and *P130* were all significantly up regulated in hypoxia during all three days of culture (B). The expression of *P21* and *P53* were also up regulated in hypoxia but on day 1 only (B). KI67 immunocytochemistry (B) showed that the fraction of KI67 positive cells (C) as well as *KI67* gene expression (B) did not differ between the two culture conditions. Scale bar: 50 μm. Filled symbols: 1% O_2_; Empty symbols: 21% O_2_. Significance level: * p<0.05, ** p<0.01.

The effect of 1% O_2_ on the expression of cell cycle related genes, *KI67*, *CCND1 (cyclin D1)*, *P21*, *P27*, *P130* and *P53* is shown in Fig 2A. As expected, KI67 was up regulated when cells were activated from G_0_-arrest, however there was no difference in the expression level at 1% O_2_ compared to 21% O_2_. The protein expression of KI67 was also studied by immunocytochemistry (Fig 2B) and the fraction of KI67 positive cells was determined (Fig 2C). A clear up regulation of Ki67 was observed after activation from G_0_ arrest, however, in accordance with the gene expression analysis, we found no difference in the fraction of KI67 positive cells between the two conditions when cultured for three days.

The expression of *P21*, and *P53* was up regulated at day 1 in 1% O_2_ compared to 21% O_2_, while the expression of *P27*, *CCND1* and *P130* was induced for all three days in 1% O_2_. Thus, low oxygen tension seemed to increase the expression of both negative and positive regulators of cell cycle kinetics, but the resulting induction in long-term proliferation suggests that low oxygen level has a positive effect on cell cycle progression.

### The effect of low oxygen tension on PAX genes and MRFs

**Fig 3:**
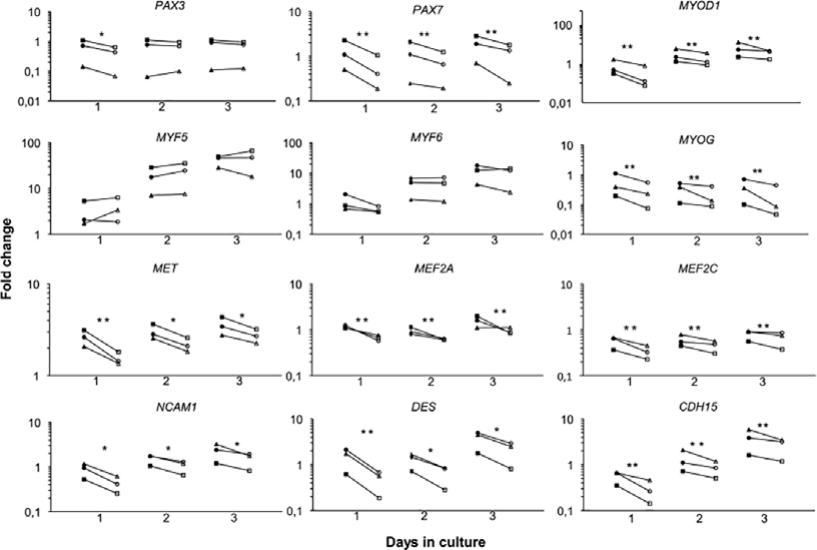
The expression of *PAX3*, *PAX7*, MRFs and genes involved in myogenesis were studied in human myoblasts re-activation from G_0_-arrest in hypoxic and normoxic culture conditions. The up regulation of *PAX7* was highly significant in 1% O_2_ for all three days in hypoxic conditions, while *PAX3* was increased only on day one. *MYOD* and *MYOG* were up regulated in 1% O_2_, whereas *MYF5* and *MYF6* had similar expression levels at 1% O_2_ and 21% O_2_. The myogenesis related genes *C-MET*, *MEF2A*, *MEF2C*, *NCAM*, *DES* and *CDH15* all had increased expression levels in 1% O_2_. Thus, most of the genes involved in the myogenic programme were induced at 1% O_2_. Filled symbols: 1% O_2_; Empty symbols: 21% O_2_. Significance level: * p<0.05, ** p<0.01.

**Fig 4:**
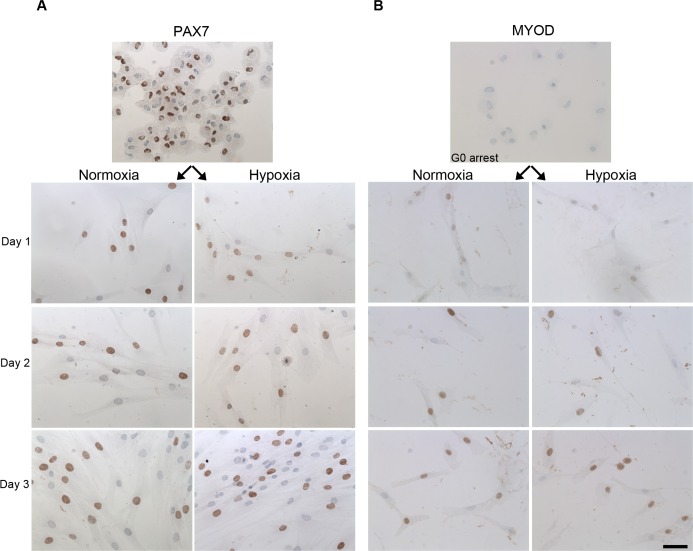
Immunostainings for PAX7 and MYOD were performed on myoblasts activated from G_0_ arrest in 1% O_2_ and 21% O_2_. We observed an up regulation of MYOD when myoblasts were activated from G_0_ arrest. However, no difference in the number of PAX7 and MYOD positive cells were found in 1% O_2_ compared to 21% O_2_. Scale bar: 50 μm.

The expression of *PAX3*, *PAX7*, MRFs and other myogenesis related genes were studied in order to see if low oxygen tension induced changes in the myogenic program when myoblasts were activated from G_0_ arrest (Fig 3). 1% O_2_ induced up regulation of *PAX3* on day 1 after activation, while *PAX7* was 2-fold up regulated throughout all three days at 1% O_2_ compared to 21% O_2_. In addition, protein expression of PAX7 was studied by immunocytchemistry (Fig 4A), however we found no difference in PAX7 protein expression in 1% O2 compared to 21% O2.

The gene expression of the early activation marker *MYOD* was more than 2-fold up regulated throughout all three days at low oxygen tension and immunocytochemical stainings verified an up regulation of MYOD during activation from G_0_ arrest (Fig 4B). The fraction of MYOD positive cells was determined (data not shown), but we found no difference in the number of MYOD positive cells in 1% compared to 21% O_2_.

Low oxygen tension induced no significant changes in *MYF5* expression, but the late myogenic marker *MYOG* (myogenin) was 2-4-fold up regulated at low oxygen tension (all 3 days). No O_2_-induced changes in *MYF6* expression were observed.

### Low oxygen tension enhance the expression of myogenic genes in human myoblasts

To further elucidate how low oxygen tension affected the myogenic lineage during activation from G_0_ arrest, we studied the expression of *cMET*, *MEF2A*, *MEF2C*, *NCAM*, *DES* (desmin) and *CDH15* (*M-cadherin)*. Low oxygen tension significantly up regulated the expression of *cMET*, *NCAM* and *DES* on day 1, while *MEF2A*, *MEF2C* and *CDH15* were up regulated all three days at 1% O_2_ (Fig 3).

**Fig 5:**
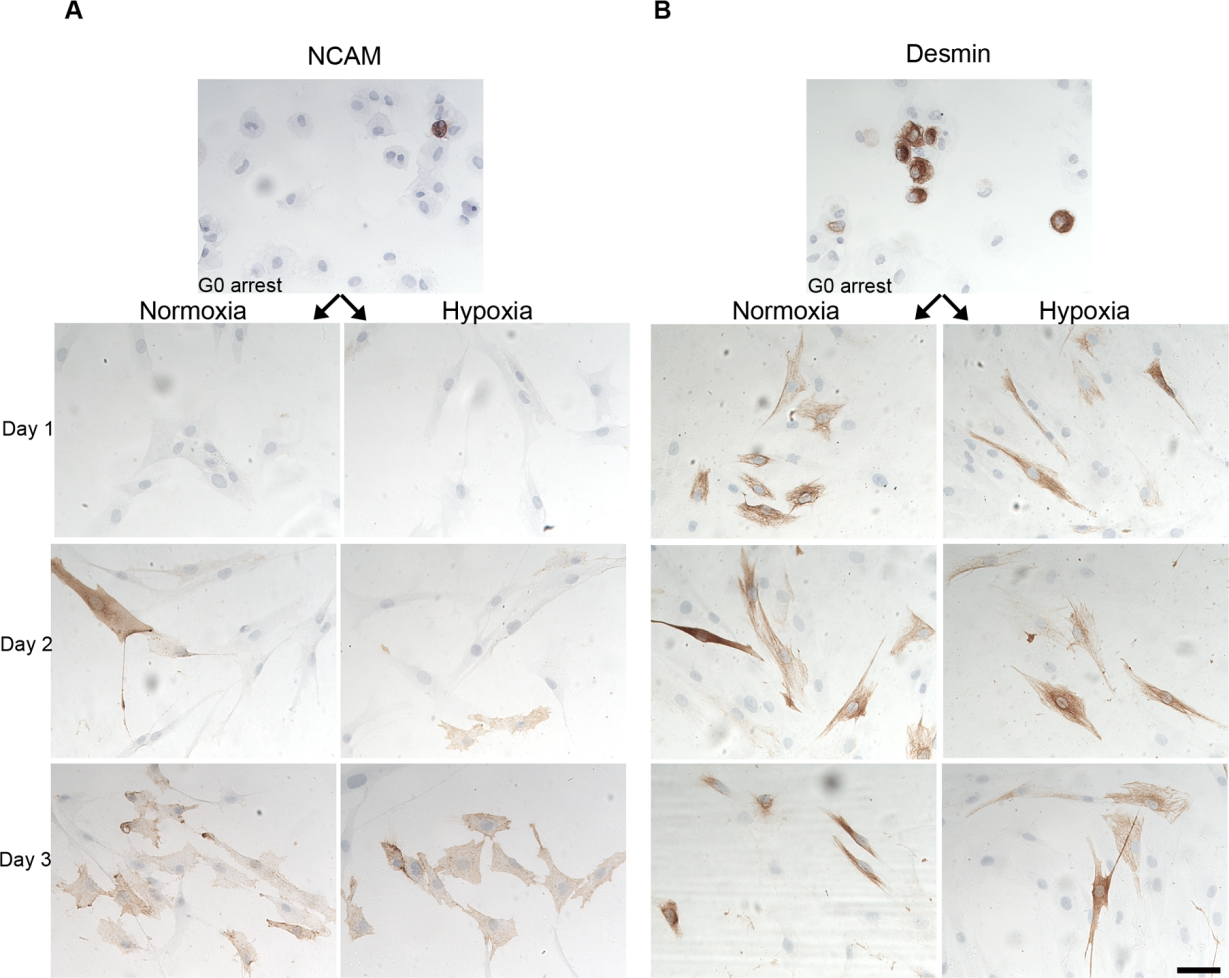
Immunostainings for NCAM and DES were performed on myoblasts activated from G_0_ arrest under hypoxic and normoxic conditions. We found large number of cells expressing both NCAM and DES, however, when quantitating the fraction of NCAM and DES positive cells, we found no consistent result pointed towards an up regulation in 1% O2, as seen in the *NCAM* and *DES* gene expressions. Scale bar: 50 μm.

The protein expression of NCAM and DES was further studied by immunocytochemistry, and representative images are shown in Fig 5. The fraction of NCAM and DES positive cells were estimated, but no differences in number of NCAM and DES positive cells were observed in conditions of 1% O_2_ compared to 21% O_2_ (data not shown).

### *HIF1A* was down regulated in myoblasts at low oxygen tension

**Fig 6:**
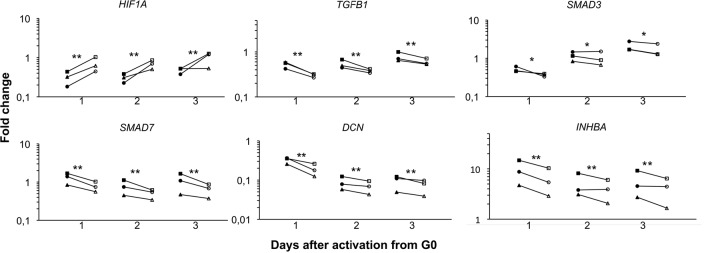
The expression of *HIF1A* was down regulated in myoblasts cultured in 1% O_2_ compared to 21% O_2_. The genes *TGF-β1*, *SMAD3*, *SMAD7*, *DCN* and *INHBA* are all part of the TGF-β signalling pathway and were all up regulated in myoblasts cultured in 1% O_2_ for all three days. Filled symbols: 1% O_2_; Empty symbols: 21% O_2_. Significance level: * p<0.05, ** p<0.01.

Hypoxia inducible factor 1A (HIF1A) signaling pathway is a major pathway for hypoxia-induced responses. Thus phosphorylated and activated HIF1A mediate transcription of many target genes involved in cell proliferation, survival and differentiation [21,22]. In contrast to some previous findings, we found that *HIF1A* gene expression was down regulated at low oxygen tension (Fig 6) during all three days.

### Effects of low oxygen tension on the TGF-β signaling pathway

The TGF-β signaling pathway is involved in numerous aspects of skeletal muscle development and regeneration. TGF-β1 is a negative regulator of myogenesis and inhibitor of muscle differentiation, and exerts its effect through the TGF-β signaling pathway [23–25]. Further, TGF-β signaling has been shown to participate in regulating the hypoxia-induced responses [26–28]. We have studied the expression of *TGF-β*, *SMAD3*, *SMAD7*, *DCN and INHBA* and found that they were all slightly, but significantly induced when myoblasts were cultured in low oxygen tension (Fig 6). The expression of *FST* was also studied, however no difference in expression levels was found (data not shown).

### The Notch signaling pathway was affected by low oxygen tension

**Fig 7:**
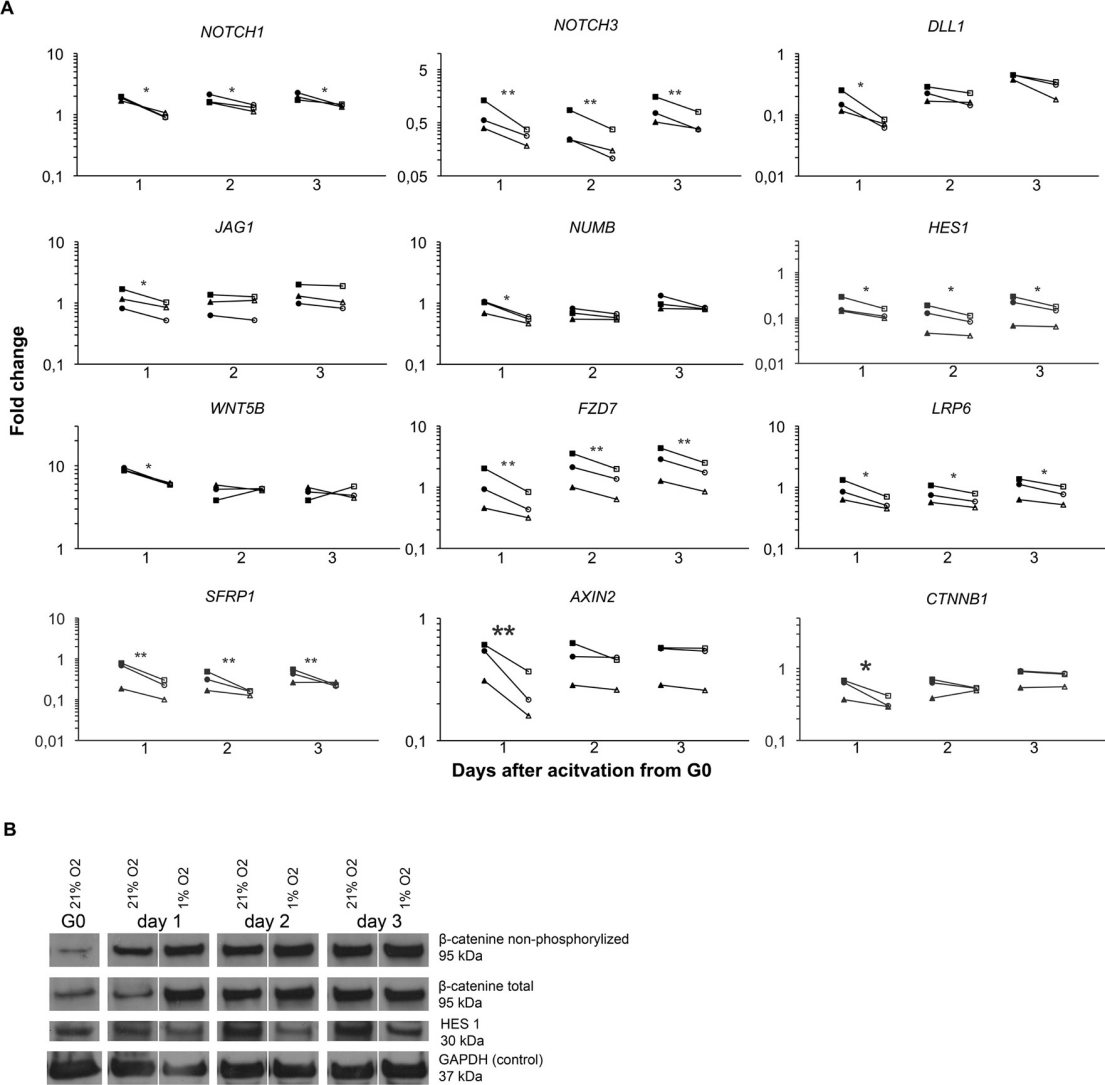
The Notch and Wnt signalling pathways were affected by hypoxic conditions when human myoblasts were activated from G_0_ arrest. The expression of *NOTCH1*, *NOTCH3* and *HES1* were up regulated in 1% O_2_ for all three days, while *DLL1*, *JAG1* and *NUMB* were up regulated on day one only. However, the HES1 protein expression was induced in 21% O_2_ on day 2 and 3. In the Wnt signalling pathway we observed an up regulation of *FZD7*, *LRP6* and *SFRP1* on day 1–3 in 1% O_2_, whereas *WNT5B*, *AXIN2* and *CTNNB1* was up regulated only on day one. Accordingly, the protein expression of total and active non-phosphorylated β-catenin was up regulated in 1% O_2_ in particular on day 1. Filled symbols: 1% O_2_; Empty symbols: 21% O_2_. Significance level: * p<0.05, ** p<0.01.

Low oxygen tension has been shown to repress differentiation in mouse myogenic cells through a Notch dependent mechanism [29] prompting it relevant to examine if Notch signaling also plays a role in activation and proliferation of human myoblasts at low oxygen tension. We found that 1% O_2_ enhanced the gene expression of the receptor *NOTCH3* 2-fold and the downstream mediator *HES1* 1.5-fold for all three days in culture (Fig 7A). In western blot (Fig 7B) the cells cultured at 21% O_2_ seemed to have a higher background signal, and when the background is subtracted, an increase in HES1 expression is seen on day 2 and 3 in 21% O_2_. Considering the background and the loading control (GAPDH), the expression of HES1 on day 1 probably does not differ in 21% O_2_ compared to 1% O_2_. The receptor *NOTCH1*, ligands *DLL1 and JAG1*, and Notch inhibitor *NUMB* were all up regulated at 1% O_2_ on day 1 only (Fig 7A).

### Effects of low oxygen tension on Wnt signaling

The canonical Wnt pathway is important for proliferation and differentiation of myoblasts [30]. To investigate if genes involved in the Wnt signaling pathway were affected by low oxygen tension, we studied the expression of *WNT5b*, *FZD7*, *LRP6*, *SFRP1*, *AXIN2* and *CTNNB1 (β-catenin)* (Fig 6) and found that these genes were all up regulated at 1% O_2_, however the up regulation in *WNT5*, *AXIN2* and *CTNNB1* was restricted to day 1. Accordingly, the amount of β-catenin (non-phosporylated (active) and total) protein was higher in cells cultured at 1% O_2_ in particular on day 1, but a small increase in of β-catenin was also seen on day 2 and 3.

## Discussion

In the present study we have demonstrated that low oxygen tension (1% O_2_) mimicking the *in vivo* tissue conditions induced not only long-term myoblast proliferation but also the expression of several genes involved in myogenesis and TGFβ, notch, and wnt signaling pathways, when myoblasts were synchronously activated from G_0_ arrest.

### Myoblast cell cycle progression was induced by low oxygen tension

The colony forming assays suggested that 1% O_2_ increased proliferation of human myoblasts, which is in accordance with a previous study made with bovine myoblast [5]. Likewise, applying 2% O_2_ tension resulted in increased proliferation rates in human myoblasts within the first 5 passages [12]. In accordance with T Launays report [3] we found no difference in proliferation during the first three days of culture in 1% and 21% O_2_. In our experience the human myoblasts have a doubling time of approximately two days, why a longer incubation time may be necessary to demonstrate a small change in proliferation rate.

In general, applying oxygen tension levels from 2–6% typically led to increased proliferation and differentiation in myoblasts obtained from various species, an oxygen level of 1% resulted in both decreased and increased proliferation rate, while further reductions in O_2_ tension to less than 1% resulted in decreased proliferation and differentiation in myoblasts obtained from humans, mice, and rats as well as in C2C12 myoblasts (Table 1). Thus, an oxygen level of 1% may be at the borderline of physiological normoxia. The gene expression study demonstrated that more genes with effect on cell cycle dynamics are sensitive to oxygen tension. However, no overall effect on proliferation could be deducted from these expression studies alone.

### The effect of low oxygen tension on the myoblasts during activation and proliferation

Induction of PAX genes and MRFs are crucial for the quiescence and activation of myogenic cells [31–35] and previous studies have reported that low oxygen tension led to changes in the expression of MRFs [4,5,11]. In accordance with these reports, we found that *PAX3*, *PAX7*, *MYOD*, *MYOG*, *cMET*, *MEF2A*, *MEF2C*, *NCAM*, *DES* and *CDH15* were all expressed at elevated levels at low oxygen tension, suggesting that the myogenic program may be enhanced during activation and proliferation from G_0_ arrest, however the fraction of DES and MYOD positive cells was not changed by low oxygen tension. Thus the protein expressions of DES and MYOD did not reflect the gene expressions in this case, and although the number of cells was increased by low oxygen tension, the myogenic potential of the cells may not be changed. In addition, the 2–4 fold increase in myogenin in low oxygen tension may be of little significance, as in myoblasts induced to differentiate, the expression of *MYOG* increased more than 600-fold (S1 Fig). Liu et al has demonstrated that 1% O_2_ tension resulted in up regulation of Pax7 in mouse myoblasts promoting self-renewal of the cultured cells [36]. Accordingly, the induction of PAX genes in low oxygen tension may indicate that low oxygen favors a more stem-like phenotype.

### HIF-1 signaling

The Hif-1 signaling is regarded as a crucial signaling pathway involved in hypoxia-induced responses [21,22]. The Hif1a subunit is markedly increased in cells in response to hypoxia [37,38], and it has also been suggested to play a role in muscle development during early embryogenesis [4,39,40]. However, *in vitro* studies (conducted at 21% O_2_) have shown conflicting results concerning Hif1-a; Wagatsuma et [41] showed a decrease in Hif1a protein during C2C12 differentiation, while Ono et al [42] showed an increase in Hif1a during differentiation and in addition, knockdown of Hif1a in C2C12 cells resulted in inhibition of differentiation. Thus, the effect of Hif1a on *in vitro* myogenesis still needs to be established.

Yun et al [4] has previously demonstrated that the Hif-1a pathway does not play an important role in the hypoxic response of C2C12 myoblasts. Likewise, our study showed that lowering the oxygen tension to 1% did not increase the expression of *HIF-1A*, suggesting that HIF-1A gene is not of functional significance for the responses seen in the present study. In addition, this finding indicates that 1% O_2_ may not represent the functional hypoxic environment experienced by human myoblasts *in vivo*. However, studies should be conducted to examine the amount of activated HIF-1A protein complex present during varying levels of O_2_ in order to fully elucidate the role of HIF-1A signaling in human myoblasts.

### Effect of low oxygen tension on TGF-β, Notch and Wnt signaling pathways

The present experiments addressed the influence of low oxygen tension on genes involved in the TGF-β, Notch and Wnt signaling pathways. The greatest effect was observed upstream in the TGF-β signaling pathway, where *TGF-β1*, *SMAD3*, *SMAD7*, and *DECORIN* were induced. TGF-β1 and SMAD3 activate and promote TGF-β signaling, while SMAD7 and DECORIN both are inhibitors. However, whether these changes result in an overall activation or inhibition of the TGF-β signaling pathway needs to be clarified in future protein expression studies. Co-immunoprecipitation studies have shown that Smad3, being a mediator of TGFβ signaling, physically associates with Hif1a and regulates gene expression [26,27]. Whether this association is responsible for the induction of the myogenic genes in the present cell culture experiments remains speculative and warrants further examination.

Several findings have suggested that activation of notch signaling promotes the expansion of myoblasts and prevent differentiation [43–45]. We found increased gene expression of Notch receptors, ligands and the notch target gene *HES1* in 1% O_2_, however the protein expression of HES1 was decreased in 1% O_2_. Thus, the increased HES1 protein expression in myoblast cultured at 21% O_2_ may explain the down regulation of the myogenic genes (e.g. *MYOG*, *DES*, *MEF2A* and *MEF2C*), however the ability of the notch signaling pathway to promotes expansion of myoblast seemed to be independent of HES1 expression as we observed larger myoblast colonies in 1% O_2_.

Contradictory roles has been assigned to Wnt/B-catenin in regulating skeletal muscle regeneration [46], however our results are in support of a study by Otto A. et al. suggesting that Wnt signaling induces the proliferation of myoblast [30]. We show that 1% O_2_ lead to an up regulation of B-catenin in myoblasts, which together with the ability to form larger colonies suggest that low oxygen tension may increase proliferation through the canonical Wnt dependent pathway.

Notably, Notch and Wnt signaling pathways were mainly affected in myoblasts on day 1 after activation from G_0_-arrest at low oxygen tension, suggesting the initial phase of myoblast activation to be most sensitive to reduced oxygen tension.

### Low oxygen tension and satellite cells

Knowledge on the significance of oxygen tension on myoblast proliferation is needed when intending to define physiological and hypoxic stress conditions for *in vitro* cell culture experiments. Moreover, the *in vitro* production of cell material for regenerative purposes might also benefit from adjustment to physiological tissue conditions such as low oxygen tension. The satellite cell response to transient exposure to hypoxia has also recently been addressed in relation to human exercise training. Thus, acute hypoxia achieved by blood flow restricted exercise (BFRE), has been shown to result in marked longitudinal gains in muscle size, strength and endurance [47–50] and a recent study by Nielsen et al has reported that BFRE can result in a marked hyper-activation and proliferation of myogenic satellite cells, resulting in significant myonuclei accretion and large gains in myofiber size (+35–45%).

In the present study we demonstrated that low oxygen tension improves the proliferative capacity of human primary myoblasts. Moreover, when using an experimental set-up for studying synchronous activation of primary isolated human myoblasts, a broad range of genes related to muscle regeneration are up regulated. Also, we could demonstrate that some genes were only induced initially indicating that these genes are sensitive to a switch from 21% O_2_ to 1% O_2_ rather than a long-term effect of the low oxygen tension itself.

The positive effect of low oxygen tension on the proliferative capacity of human myoblasts makes further studies in the field interesting. We have already demonstrated that many pathways are influenced by the oxygen tension but many aspects remain to be explored in order to elucidate the effect on the activation and proliferation of human myoblasts. For future directions, gene expression as well as protein expression studies will be useful in order to understand the cellular mechanisms that are targeted by hypoxic conditions. Furthermore, it would be of interest to determine if low oxygen tension influences the quiescent state of satellite cells, and thus could be an important parameter in satellite cell activation, as suggested by recent longitudinal data [51]. In relation to this notion, it will also be of interest to investigate if the severe, transient hypoxia experienced during acute occlusion training (BFRE) exerts its effect due to direct influence on the pool of myogenic satellite cells or acts via a mechanism activated by the muscle fibers and/or vascular epithelial cells.

## Materials and Methods

### Ethics statement

The muscle biopsies were obtained from three healthy human donors; two men (vastus lateralis) and one woman (gluteus maximus) aged 18–20 years. The muscle biopsies had a weight of 50–100 mg. The participants gave written informed consent and the local ethics committee of Region of Southern Denmark (S-20070079) approved the study.

### Isolation and propagation of human myoblasts

All experiments described in this study were made with myoblasts isolated from three healthy donors. The human primary myoblast cultures were established as previously described [14,52]. Biopsies free of connective tissue were minced and digested with 0.3% collagenase type II (Medinova Scientific) for 40 min. in 37°C water bath. The suspension was triturated with a 1 ml pipette, cold HBSS (Hanks Balanced Salt Solution) with 10% FBS was added and the suspension was pelleted and re-suspended in 37°C HBSS with 10% fetal bovine serum (FBS) and filtrated first through a 100 μm then through a 40 μm Falcon Cell Strainer. The isolated satellite cells were cultured in GM and plated on extracellular matrix (ECM, Sigma-Aldrich) coated dishes (Nunclon, Nunc). During every passage, the number of fibroblasts was reduced by pre-plating the cells for 20 min. at 37°C on untreated NUNC dishes and non-adherent cells were harvested and expanded at 21% O_2_ for three passages and aliquots were frozen and kept in liquid nitrogen.

### Growth rate assessment

Proliferating human myoblasts (3rd passage) were seeded in 6-well plates (Nunclon, Nunc) with a density of approx. 1560 cells/cm^2^. Half of the plates were placed in humidified incubator (37°C) at 21% O_2_ and the other half was placed at 1% O_2_. After 1, 2 and 3 days the cells were detached with trypsin-EDTA and counted using a hemocytometer. The experiments were made in triplicates.

### G_0_ arrest and reactivation of human myoblasts

The procedure for G_0_ arresting human myoblasts was modified from previous studies [14,18]. Briefly, proliferating human myoblasts (5th passage) were detached with Trypsin-EDTA, pre-plated and transferred to suspension medium, SM, (DMEM with 2% methyl cellulose (Sigma-Aldrich), 10% FBS and 1% PS) and cultured in dishes with ultra low attachment surface (Corning) with a density of 150.000 cells/ml at 21% O_2_. This suspension culture condition makes the cells enter G_0_ arrest.

For re-activation of cells from the suspension medium, cells were washed twice by dilution in PBS followed by centrifugation. The pellet was re-suspended in lysis buffer (for RNA isolation), cytospun (for immunocytochemistry) or plated on ECM-coated dishes or coverslips (Thermanox, Nunc). Re-activated myoblast cultures were harvested for RT-qPCR and immunocytochemical studies on day 1, 2 and 3. Myoblasts were differentiated for 5–7 days in DMEM with 2% FBS, 1% PS and 25 pmol Insulin (Actrapid from Novo Nordisk).

### Clonal assay and crystal violet staining

Human myoblasts were seeded in cell culture dishes (Nunc) with a cell density of 4,6/cm^2^ and cultured in 1% and 21% O_2_ tension, respectively. After 14 days of culture, the cells were rinsed in PBS, fixed in 4% NBF and staining with 0.05% crystal violet staining solution for 30 min followed by wash in tap water and left to dry. The number of colonies was counted.

### Immunocytochemistry

For immunocytochemical analyses, cells cultured on coverslips in GM were rinsed twice in PBS and mounted on glass slides.

G_0_ arrested cells were washed as previously described and resuspended in PBS and loaded on Shandon cytofunnel (Thermo Scientific) and centrifuged on SuperFrost^®^ Microscope Slides with Shandon Cytospin 4 Cytocentrifuge.

For detection of desmin and NCAM, samples were fixed in acetone, 10 min., followed by addition of mouse-anti-desmin, D-ER-11 (Dako) 1:25 or mouse-anti-NCAM Leu19 (BD Biosciences) 1:50. EnVision (Dako) was used as detection system and DAB as chromogen.

For detection of MyoD, samples were fixed in 4% NBF, 5 min., followed by addition of mouse-anti-MyoD1, 5.8A (Novocastra). For detection of Pax7, the cells were fixed in 4% NBF for 15 min., 96% ethanol for 10 min. followed by heat induced epitope retrieval in TEG buffer (95°C) for 15 min. and incubation in mouse-anti-Pax7 (Developmental Studies Hybridoma Bank). PowerVision (Dako) was used as detection system and DAB as chromogen for MyoD and Pax7 stainings.

For detection of KI67, samples were incubated in 4% NBF for 15 min. followed by incubation in 96% ethanol for 10 min. Samples were then rinsed in water before heat-induced epitope retrieval for 15 min in Tris-EGTA buffer, PH 9,0 at 95 ˚C, followed by addition of mouse-anti-KI67, MIB1 (Dako). EnVision (Dako) was used as detection system and DAB+ as chromogen.

Nuclei were counterstained with Mayers Hemalum.

### Morphometric analyses

CAST version 2.1.6.0 (Visiopharm) was used for all morphometric analysis. Random counts were performed including the entire cell containing area. KI67, NCAM and desmin positive cells and total number of cells were counted in 10% of the area and at least 200 cells were counted for each specimen. The identity of all samples was blinded.

### RT-qPCR analysis

Cells cultured in GM were rinsed twice in PBS and lysed in 1x Nucleic Acid Purification Lysis Solution (Applied Biosystems). Cells cultured in SM were washed as previously described and lysed with Lysis Solution. RNA was isolated using ABI PRISM^TM^ 6100 Nucleic Acid PrepStation with Total RNA Chemistry kit (Applied Biosystems) according to manufacturer’s instruction/protocol.

cDNA was generated from 1000 ng of isolated total RNA using High Capacity cDNA Reverse Transcription Kit (Applied Biosystems). qPCR was performed on ABI Prism 7900HT Sequence Detection System using Taqman Array platform (Applied Biosystems). Taqman Arrays were customer designed 384-well micro fluidic cards containing 32 genes including 4 reference genes. In this study 2 different sets of arrays were used. Array 1 contained the genes: *18S*, *CDH15*, *DCN*, *DES*, *FST*, *HIF1A*, *INHBA*, *MEF2A*, *MEF2C*, *MET*, *MSTN*, *MYF5*, *MYF6*, *MYOD1*, *MYOG*, *NCAM1*, *PAX3*, *PAX7*, *PGK1*, *SMAD3*, *SMAD7*, *TBP*, *TFRC*, *TGFB1*. Array 2 contained the genes: *18S*, *AXIN2*, *CYCLIN D1*, *CDKN1A (P21)*, *CDKN1B (P27)*, *CTNNB1*, *DLL1*, *DLL3*, *FZD7*, *HES1*, *JAG1*, *LRP6*, *MKI67 (KI67)*, *NOTCH1*, *NOTCH3*, *NUMB*, *PGK1*, *RBL2 (P130)*, *SFRP1*, *TBP*, *TFRC*, *TP53 (P53)*, *WNT1*, *WNT3A*, *WNT5B*. Of these, *DLL3*, *WNT1*, *WNT3* and *MSTN* had missing values and could not be analyzed. Gene expression data for *ADAM12*, *DLK1*, *FGF2*, *FGF8*, *FGFR1*, *IGFR1*, *MYH8*, *SPARC*, *CASP3*, *DKK1*, *MLL5*, *NUPR1*, *PCNA*, *PRDM2* and *S100A4* are not shown. All assays were run in triplicates and the experiment series was made with cells from three individuals (3 biological replicates). Raw data was retrieved using the SDS 2.1 software, analyzed with automatic threshold settings and the Cq values were exported to qbase^PLUS^ software (Biogazelle). The most stable reference genes were selected by exporting the Cq values of all four reference genes (*18S*, *TBP*, *PGK1* and *TRFC*) to the software geNorm version 3.5 where the gene expression normalization factor for each sample based on the geometric mean of the reference genes was calculated [53]. The reference genes were selected based on the gene expression stability measure M for a reference gene as the average pair wise variation V for that gene with all other tested reference genes. Based on these calculations *TBP* and *TFRC* were selected as reference genes for array 1, while *18S*, *TBP* and *TRFC* were selected as reference genes for array 1. Relative quantification was made using qbase^PLUS^ v.1.1 software [54]. The triplicates were allowed to differ by 0.5 Cq.

### Western blot

Cell samples were washed twice in PBS. Total protein was extracted by re-suspending cell pellets in RIPA buffer containing 1x Halt Protease Inhibitor Cocktail (Thermo Scientific) and 1x Halt phosphatase Inhibitor Cocktail (Thermo Scientific) followed by incubation on ice for 30 min. The lysate was centrifuged (15 min, 12,000xg, 4°C) and the supernatant was stored at -80°C until use. Protein concentration was determined using Pierce® BCA protein assay kit (VWR).

For analysis, app. 10 μg of protein was added loading buffer (Thermo Fischer Scientific) and sample reducing agent (Thermo Fischer Scientific), heated to 95°C for 5 min and loaded onto 4–12% Bis-Tris gels (Thermo Fischer Scientific). The gel was run using 1xMES buffer (Thermo Fischer Scientific) for 1 h at 120 V constant and electroblotted unto 0.45μm PVDF membranes (Millipore). The membranes were blocked with 5% skimmed milk/TBS-T for 1 hour at room temperature and incubated overnight at 4°C with the following primary antibodies: rabbit-anti-human HES1 (Cell Signaling Technology, #11988) 1:1000, rabbit-anti-human β-Catenin total (Cell Signaling Technology, #9562) 1:1000, rabbit-anti-human β-Catenin non-phosphorylated (Cell Signaling Technology, #19807) 1:1000, or rabbit-anti-human GAPDH (Santa Cruz Biotechnology, #sc-25778). The following day blots were washed in TBS-T (3X), and incubated for 1 hr at room temperature with goat-anti-rabbit-HRP (DAKO, p0448) 1:2000, washed in TBST (3X) and developed using Novex ECL HRP Chemiluminescent Substrate kit (Thermo Fischer Scientific) and standard x-ray film. GAPDH was used as loading control.

### Statistical analysis

Statistical analyses were performed using Stata 10.1 (StataCorp. 2007; *Stata Statistical Software*: *Release 10*; StataCorp LP, College Station, TX, USA). Gene expression in G_0_-arrested cells was set as baseline and the expression in 21% O_2_ was compared to 1% O_2_ for day 1, 2 and 3 using a linear mixed-effects model with subjects as random effects and with time (day 1, 2 and 3) and group (1% O_2_ versus 21% O_2_) as fixed effects. Model assumptions about normal distribution of residuals and homogeneity of variance were satisfied by log-transformation of data. Significance level was set at a = 0.05.

## Supporting Information

S1 Fig Expression of *MYOG* in G_0_ arrested, proliferating and differentiated human myoblasts. A very low expression of *MYOG* gene is detected in G_0_ arrested and proliferating myoblast, however the expression is more than 600-fold increased in differentiated cells.
(TIF)
