# Supplementary figures and images for: Low Oxygen Tension Enhances Expression of Myogenic Genes When Human Myoblasts Are Activated from G0 Arrest
Source: PLoS One. 2016 Jul 21;11(7):e0158860. doi: 10.1371/journal.pone.0158860 (PMC4956100; doi:10.1371/journal.pone.0158860)

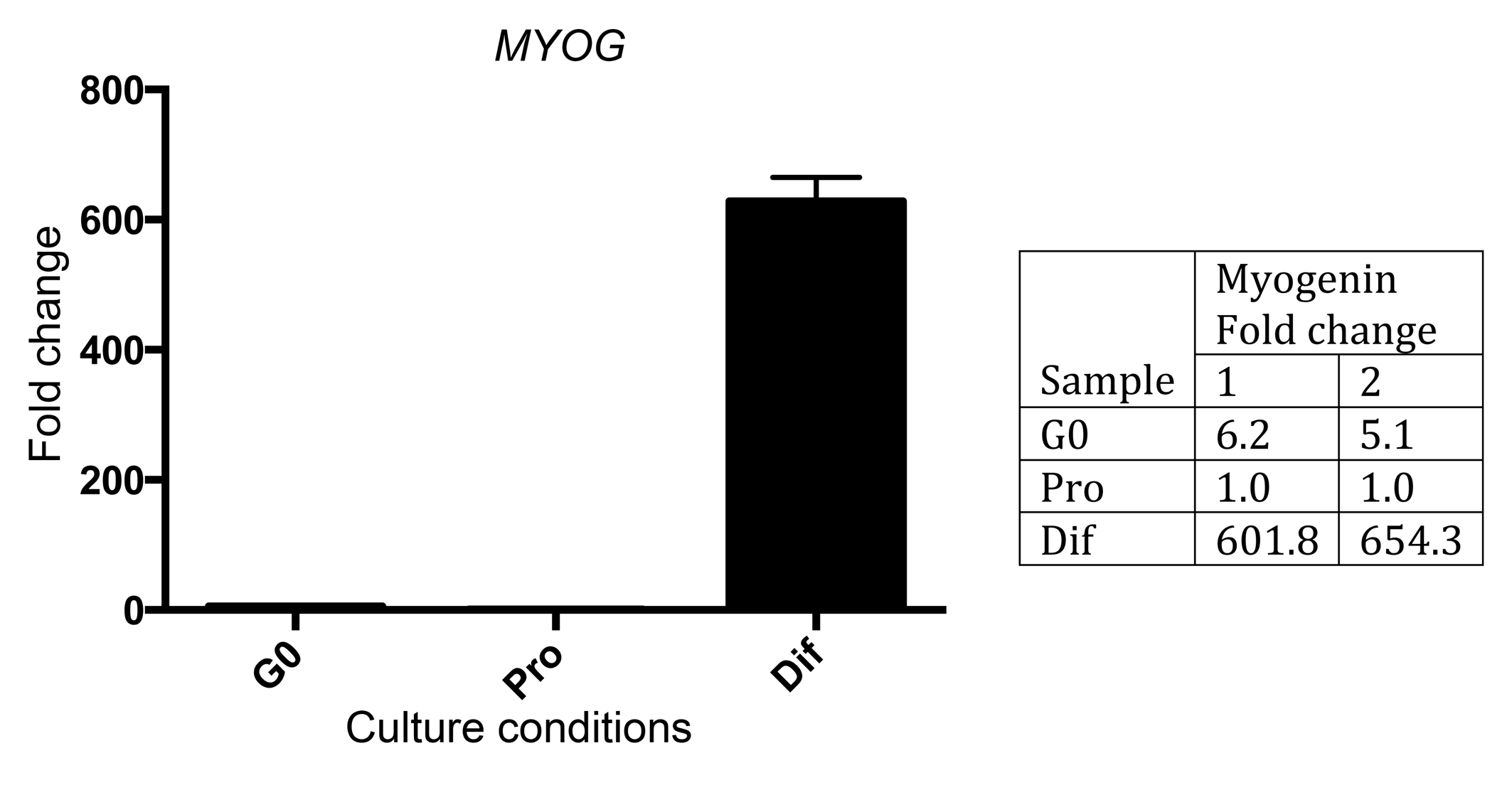

Supplement: S1 Fig — A very low expression of MYOG gene is detected in G0 arrested and proliferating myoblast, however the expression is more than 600-fold increased in differentiated cells. (TIF) [file pone.0158860.s001.tif]
